# Alteration of immunoregulatory genes expression in mesenchymal stromal cells upon priming with B18R as an interferon binding protein

**DOI:** 10.22038/IJBMS.2022.67353.14771

**Published:** 2023-02

**Authors:** Hamid Reza Bidkhori, Moein Farshchian, Mahboubeh Kazemi Noughabi, Halimeh Hassanzadeh, Houshang Rafatpanah

**Affiliations:** 1 Immunology Research Center, Inflammation and Inflammatory Diseases Division, Mashhad University of Medical Sciences, Mashhad, Iran; 2 Stem Cells and Regenerative Medicine Department, Academic Center for Education, Culture, and Research (ACECR)-Khorasan Razavi, Mashhad, Iran; 3 Department of Biology, Faculty of Science, Ferdowsi University of Mashhad, Mashhad, Iran

**Keywords:** B18R, Gene expression, Immune-related genes, Interleukin, Lipopolysaccharide, Mesenchymal stromal cells, poly(I:C)

## Abstract

**Objective(s):**

The B18R protein encoded by the Vaccinia virus decoys Type 1 interferons and inhibits the activity of several type I IFN members. In vitro transcription protocols benefit from this molecule’s involvement in enhancing cell viability by inhibiting interferon signal transduction. As a result of their immunomodulatory properties and potential to regenerate, mesenchymal stromal cells (MSCs) are increasingly considered an alternative treatment for a wide range of immune disorders. In this study, we investigated the modification of expression of several genes involved in immune-related pathways after preconditioning MSCs with two immune stimuli, including poly(I:C) and LPS.

**Materials and Methods:**

ASCs were isolated and primed with B18R, and after exposure to poly(I:C) and LPS, the expression of the same sets of genes as in the previous experiment was evaluated. Following total RNA isolation from primed cells and cDNA preparation, real-time quantitative PCR was performed for several immunomodulatory and immune-related genes, including *IDO1, TDO2, COX-2, TGF-β1, TNF-α, IL-1β, IL-6, TLR3, TLR4*, and *MCP-1*.

**Results:**

Pretreatment of MSCs with poly(I:C) and LPS significantly increased the expression of all mentioned genes, while upon the B18R challenge followed by poly(I:C) and LPS treatment, they were down-regulated. Finally, it was observed that the relative expression level of *IFN-β* has significantly decreased in MSCs+B18R+poly(I:C) and LPS in comparison with these groups without B18R.

**Conclusion:**

The data indicated that the presence of B18R prevents the overexpression of several immune-related genes, which are overexpressed in the *in vitro* inflammatory environment.

## Introduction

The antiviral response of the cell is activated after binding of interferon (IFN) type I to the interferon receptors on the cell surface, which results in the induction of IFN-stimulated genes (ISGs) transcription. Overexpression of these genes is followed by inhibiting virus replication and, finally, suppressing viral infection. One of the strategies developed in the *Vaccinia virus* to create an antiviral state is the expression of a soluble receptor for IFN type I, called B18R. This protein binds to the cell surface and prevents antiviral response by attaching to the IFNs. B18R is extensively applied after synthetic mRNA transfection and other RNA delivery technologies to increase cell viability (1, 2). As a surface antigen, this protein was originally detected on the surface of poxvirus-infected cells. However, it has recently been demonstrated to be a secreted protein (3). Cells infected with the *Vaccinia virus* express two molecular species of B18R (52 and 60 to 65 kDa ), the second of which is secreted into the culture medium. A signal peptide was observed at the N terminus of the B18R protein secreted from cells infected with the *Vaccinia virus* or recombinant baculovirus. It has been shown that virus virulence is diminished in mice lacking the B18R gene, indicating that the protein plays a role in viral virulence. As opposed to highly species-specific cellular IFN receptors, the B18R protein can bind a wide range of them, including those from rats, rabbits, and humans (4).

An immune response is triggered following the sensing of foreign RNA inside the cell through induction of Toll-like receptors (TLR) 3, 7, and 8 or retinoic acid-inducible gene I. Upon activating this defense mechanism, IFNs are released, leading to the deletion and inhibition of RNA and translation. Thus, successful delivery of the synthetic RNA requires overcoming the blockade activity of the IFNs, and this goal may be achieved by co-transfer of synthetic B18R RNA and the mRNA of interest (2). This strategy could be an excellent benefit for mRNA therapeutics in clinical translation.

Moreover, the immune evasion ability of B18R makes it a favorable molecule in other clinical applications. One of the strategies used in cancer virotherapy is attenuating the innate immune response to the virus, which can predominantly increase the anti-tumor effects. IFNs are considered one of the main components of the innate antiviral immune response with a fundamental role in controlling infection. To suppress the inhibitory effects of IFN, incorporating the *Vaccinia virus* *B18R* gene into the oncolytic virus genome blocks the innate immune response and potentiates the anti-tumor effects (5). 

Mesenchymal stromal cells (MSCs) are remarkable resources for clinical applications such as immunomodulation and regenerative cell therapy. There are two sides to regulating the immune response controlled by MSCs, and it depends on the environmental condition. For example, the immunosuppressive activity of MSCs is mediated in the presence of pro-inflammatory cytokines through overexpression of indoleamine-pyrrole 2,3-dioxygenase (IDO), prostaglandin E2 (PGE2), and cyclooxygenase 2 (COX-2). Furthermore, MSCs primed with IFN-γ in graft-versus-host disease (GvHD) become as effective at suppressing the immune system as possible. MSCs are needed to be tuned for a particular clinical condition. The administration of MSCs without any modulation to treat various clinical conditions is not optimized (6, 7). One favored strategy to enhance MSCs’ stromal potential is pretreatment by biological and biochemical factors. MSCs are primed in the culture media to boost their therapeutic properties by adding cytokines, chemicals, biomaterials, hypoxia, and other molecules, including cytokines or chemicals (8).

In addition to many studies reporting the leading role of MSCs in treating immune-related complications, there are some investigations on intrinsic MSCs defects caused by over-activity of the immune response. For instance, bone marrow Mesenchymal Stromal cells (BMSCs) in systemic lupus erythematosus (SLE) patients are abnormal with a low capacity for immunomodulatory and *in vitro* growth and proliferation. The evidence has shown that high IFN-β production in SLE BMSCs leads to chronic inflammation and, eventually, an impaired cell cycle. Although they keep releasing IFN-β and other inflammatory factors, cellular senescence occurs (9). Ruxolitinib (Ruxo), as a JAK pathway inhibitor, is an approved drug for Philadelphia-negative chronic myeloproliferative neoplasms (MPNs). In MPN, overproduction of pro-inflammatory cytokines in the bone marrow stroma leads to disease pathology, including bone marrow fibrosis. BMSCs contributing to this condition were studied as targets of Ruxo (10).

To the best of our knowledge, there are no previous studies on the effects of B18R on the immunological properties of MSCs and any other biological process in MSCs. There is still a great obstacle to successful MSC-based therapies due to their high sensitivity to inflammatory environments, and inhospitable tissue environments can limit transplanted MSC function and survival. In this way, “empowered” primed MSCs may be more effective and have broader applications than their previous counterparts (6). Here, we investigated the expression of several genes related to the immune response in adipose-derived MSCs (ASCs) after treatment with B18R. The observed expression pattern may provide practical information on the application of IFN-β inhibitors, especially B18R, in priming approaches and culture conditions to improve the efficacy of MSCs for pre-clinical and clinical applications.

## Materials and Methods

***Isolation of ASCs***

Lipoaspirates were obtained from three healthy individuals who underwent liposuction surgery at a cosmetic day clinic in Mashhad. The project was approved by (ACECR-Khorasan Razavi Biomedical Research Ethics Committee Code: IR.ACECR.JDM.REC.1398.007), and informed consent was taken from all participants. The samples were washed three times with phosphate-buffered saline (PBS) containing 0.1% penicillin-streptomycin (pen-strep; Biosera, France). The samples were placed fixed for 15 min as stationary and then treated with freshly prepared 0.1% collagenase type I (Invitrogen, USA) and fetal bovine serum 10% (FBS; Gibco, USA) for 1 hr. Cell debris was then removed by centrifugation at room temperature and 600g for 10 min. The pellet was washed in PBS and centrifuged (400 g, 6 min). Finally, the cells were transferred to T75 tissue culture flasks containing Dulbecco’s Modified Eagle’s Medium (DMEM; Biowest, France) with 10% FBS and 0.1% pen-strep and maintained under a 5% CO_2_ incubator at 37 °C. Once cells reached a confluency of 85 to 90% at a density of 10000 cells per cm^2^. In all subsequent experiments, ASCs were used at passage three (11). 

***Characterization of ASCs***

Mesenchymal lineage-specific surface markers were identified using flow cytometry (BD Accuri C6, USA). Cells were incubated with antibodies conjugated with PE for CD73, CD13, FITC for CD90, CD34, CD14, and HLA-DR, along with APC for CD45 and CD44 for 45 min ( Cytognos, Spain). The analysis was performed using the FlowJo software (version 7.6.1). ASCs were proven to be multi-lineage capable by causing them to differentiate into adipogenic and osteogenic lineages in the presence of adipogenesis and osteogenesis differentiation media for 14 days and 21 days, respectively. The adipogenic medium contained DMEM supplemented by 10% FBS, 200 mM indomethacin, 1 mM dexamethasone, and 10 mM β-glycerophosphate (all from SigmaAldrich, Germany). The osteogenic medium also was supplemented with 50 mM ascorbate-2- phosphate (Sigma Aldrich, Germany), 10 mM β-glycerophosphate, and 0.1 mM dexamethasone. After sufficient induction time, cells were stained with oil red O (Sigma Aldrich, Germany) to visualize the lipid droplet in the adipogenic induction plate and alizarin red (Sigma Aldrich, Germany) to detect calcium mineralization in osteogenic induction media. As part of the osteogenesis investigation, an alkaline phosphatase (ALP; Sigma Aldrich, Germany) assay was used, and the enzyme activity was measured using a substrate (12).

***Treatment of ASCs***

ASCs growing to 70–80 % confluence were incubated with B18R (0.1 μg/ml, Thermo Scientific, USA) for 24 hr. Then the cells were treated with TLRs agonists. The agonists used for TLR pretreatment of MSCs were poly(I:C) (10 ng/μl; Sigma-Aldrich, USA) and lipopolysaccharide (LPS; 1 μg/ml; Sigma-Aldrich, USA). The cells were washed twice in a complete cell culture medium before being used for the following assay. There were four groups of treated cells: ASCs + poly(I:C), B18R ASCs + poly(I:C), ASCs + LPS, and B18R ASCs + LPS. For the first group, the controls were ASCs without any treatment and ASCs treated with poly(I:C), respectively. The third and fourth test groups were compared with ASCs without treatment and ASCs treated with LPS, respectively.

***Quantitative real-time PCR analysis***

To evaluate the gene expression, the cell pellets obtained from the previous experiment were subjected to total RNA isolation using Tripure reagent (Roche, Germany) according to the manufacturer’s directions. The concentration of RNA was determined (Nanodrop ND-1000 spectrophotometer; Bio-Tek, USA), and 1 μg of RNA was reverse transcribed using the PrimeScript RT reagent Kit’s instructions (TAKARA, Japan). Quantitative real-time PCR (RT-qPCR) was performed using the Bio-Rad CFX-96 system (Bio-Rad, USA). The ribosomal protein lateral stalk subunit P (RPLP0) was used to normalize the expression level of the target genes. 2^–(^^ΔΔCt^^)^ formula was used for gene expression analysis. The primer sequences applied for amplification are shown in Table 1.  Two samples t-test was used for data analysis via GraphPad Prism statistical program (version 9; San Diego, CA, USA). Data are reported as the mean of at least three independent experiments ± SEM and *P-values *less than 0.05 were considered statistically significant. 

***Statistical analysis***

Two samples t-test was used for data analysis via GraphPad Prism statistical program (version 9; San Diego, CA, USA). Data are reported as the mean of at least three independent experiments ± SEM and *P-values *less than 0.05 were considered statistically significant. 

## Results

***Characterization of human ASCs***

**Figure 1:**
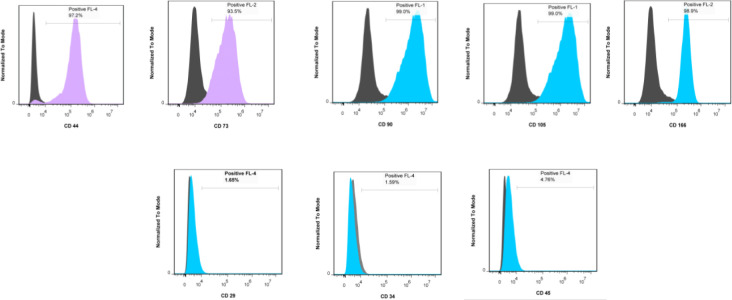
Flow cytometric characterization of ASCs. Top, markers are expressed positively (more than 93% for all selected markers). Bottom, ASCs express these negative markers

Human ASCs were investigated for the selective proliferation of MSCs by evaluating their surface markers and multi-lineage capability. ASCs exhibited higher (>93 %) expression of MSC-specific surface CD markers including CD44, CD73, CD90, CD105, and CD166 . All negative markers were under 5% (CD29, CD34, and CD45) (Figure 1). 

**Figure 2:**
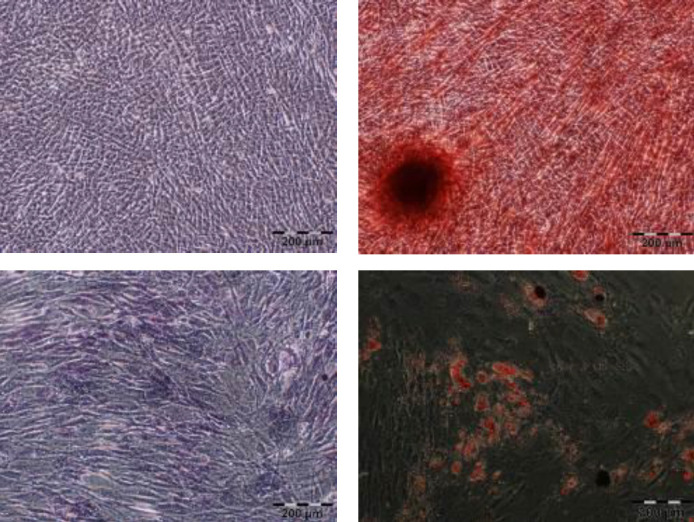
Multi-lineage mesenchymal differentiation potential of ASCs using the appropriate inductive media. Top, left: untreated ASCs as control, right: differentiation into osteocytes shown by calcium deposition upon alizarin red S staining. Bottom, left: osteogenesis was also confirmed through ALP assay, right: differentiation into adipocytes, evidenced by oil red O staining (scale bars represent 200 μm)

ASCs: Adipose-derived MSCs; ALP: Alkaline phosphatase

These adipogenic and osteogenic differentiation assays were used to determine whether isolated ASCs were capable of differentiation toward these two lines of cells. An intracellular lipid droplet staining using oil red O proved that ASCs underwent adipogenesis, and alizarin staining clearly showed the mineralization of the extracellular matrixes on the differentiated ASCs. The undifferentiated ASCs did not reveal these observations. In addition, a higher level of ALP activity was observed in the cells of the osteogenic media compared with the ASCs in the normal media, indicating osteogenic differentiation (Figure 2).

***Immune response-related genes expression in different groups of treated ASCs***

Since B18R protein has been demonstrated to have immunosuppressive activity against type I interferons, we examined its effects in ASCs upon induction of these cells with innate immune stimuli. Poly(I:C) and LPS were used to stimulate TLR3 and TLR4 on ASCs pretreated with B18R, and Real-time PCR quantified the expression of immunomodulatory and inflammatory cytokines in different groups of studied cells.   

**Figure 3:**
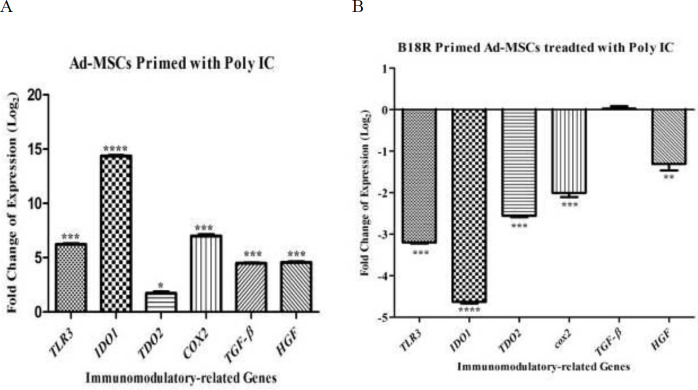
Relative expression level of immunomodulatory-related genes, A: after Ad-MSCs were treated with poly(I:C), B: after B18R primed Ad-MSCs were treated with poly(I:C) (normalized with RPLP0). The controls for the first and second groups are Ad-MSCs without any treatment and Ad-MSCs treated with poly IC, respectively. (*P-value<0.05, ** *P*-value<0.01, *** *P*-value<0.001, and **** *P*-value<0.0001)

MSCs: Mesenchymal stromal cells; RPLP0: Ribosomal protein lateral stalk subunit P

**Figure 4:**
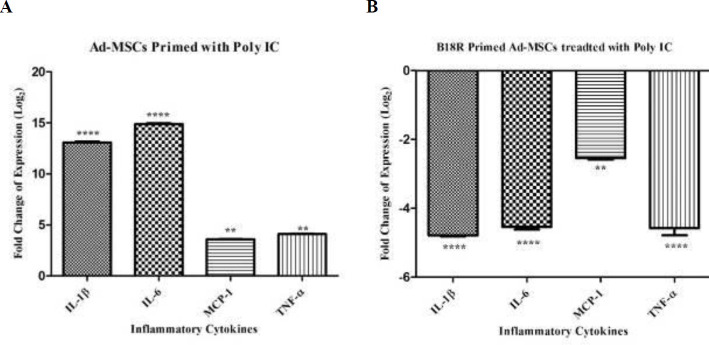
Relative expression level of inflammatory cytokine genes, A: after Ad-MSCs were treated with poly(I:C), B: after B18R primed Ad-MSCs were treated with poly(I:C) (normalized with RPLP0). Controls for the first and second groups are Ad-MSCs without any treatment and Ad-MSCs treated with poly(I:C), respectively (** *P*-value< 0.01 and **** *P*-value<0.0001)

MSCs: Mesenchymal stromal cells; RPLP0: Ribosomal protein lateral stalk subunit P

The mRNA expression of *TLR3*, *IDO1*, *TDO2*, *COX2*, *TGF-β1,* and *HGF* was significantly increased in the activated Ad-MSCs by poly(I:C) compared with control gene *RPLP0* (*P*<0.001, *P*<0.0001,*P*<0.05, *P*<0.001 *P*<0.001, and *P*<0.001, respectively) (Figure 3A). Activation of B18R primed Ad-MSCs by poly(I:C) significantly decreased the mRNA expression of *TLR3*, *IDO1*, *TDO2*, *COX2*, and *HGF* compared with *RPLP0* (*P*<0.001, *P*<0.001,*P*<0.001, *P*<0.001, and *P*<0.01, respectively), however, there were no significant differences in the *COX2*. mRNA expression between the Ad-MSCs and B18R primed Ad-MSCs (Figure 3B) *IL1-β*, *IL6*, *MCP1*, and *TNF-α* are other inflammatory cytokines examined at the mRNA level in this study. The same expression pattern as the immunomodulatory genes was found for these genes (Figure 4A, B).

**Figure 5:**
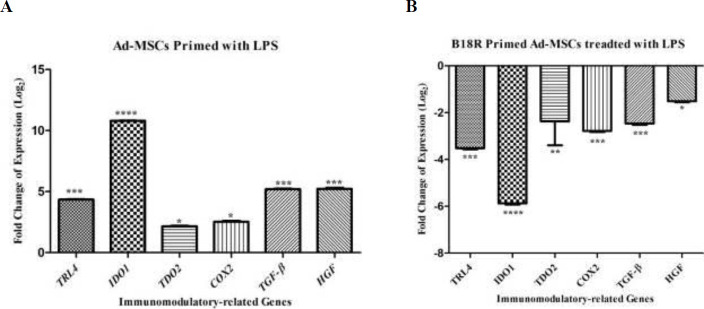
The relative expression level of immunomodulatory-related genes, A: after Ad-MSCs were treated with LPS, B: after B18R primed Ad-MSCs were treated with LPS (normalized with RPLP0). The controls for the first and second groups are Ad-MSCs without any treatment and Ad-MSCs treated with LPS, respectively (*P-value<0.005, ** *P*-value<0.01, *** *P*-value<0.001, and **** *P*-value<0.0001)

MSCs: Mesenchymal stromal cells; RPLP0: Ribosomal protein lateral stalk subunit P; LPS: lipopolysaccharide

**Figure 6:**
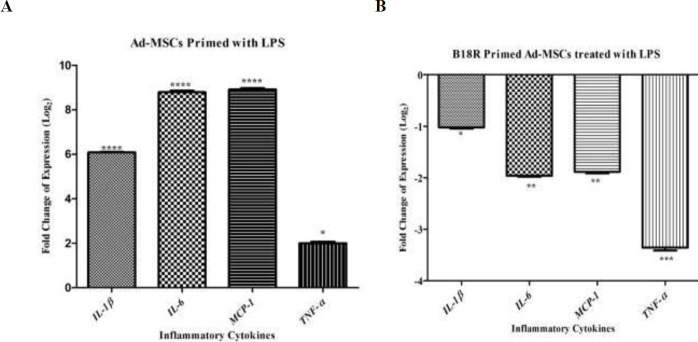
Relative expression level of inflammatory cytokine genes. A: after Ad-MSCs were treated with LPS, B: after B18R primed Ad-MSCs were treated with LPS (normalized with RPLP0). Controls for the first and second groups are Ad-MSCs without any treatment and Ad-MSCs treated with LPS, respectively (*P-value<0.005, **P-value<0.01, *** *P*-value <0.001, and **** *P*-value<0.0001)

MSCs: Mesenchymal stromal cells; RPLP0: Ribosomal protein lateral stalk subunit P; LPS: lipopolysaccharide

The mRNA levels of *TLR4*, *IDO*, *TDO2*, *COX2*, *TGF-β1,* and *HGF* were significantly increased in the activated Ad-MSCs by LPS compared with control gene *RPLP0* (*P*<0.05)(Figure 2A), while activation of B18R primed Ad-MSCs by LPS substantially decreased the mRNA levels of the above factors (*P*<0.05) (Figure 5A, B). Furthermore, activation of Ad-MSCs by LPS showed significantly enhanced *IL1-β*, *IL6*, *MCP1*, and *TNF-α* production, while the levels of these cytokines substantially decreased in B18R primed Ad-MSCs (*P*<0.05)(Figure 6A, B). 

***Decrease of IFN-β expression upon poly(I:C) and LPs treatment***

Expressions of *IFN-β* were normalized with *RPLP0* as the interior control gene in ASCs + poly(I:C) and ASCs + LPS groups, and also in similar groups which were treated with B18R. 

**Figure 7:**
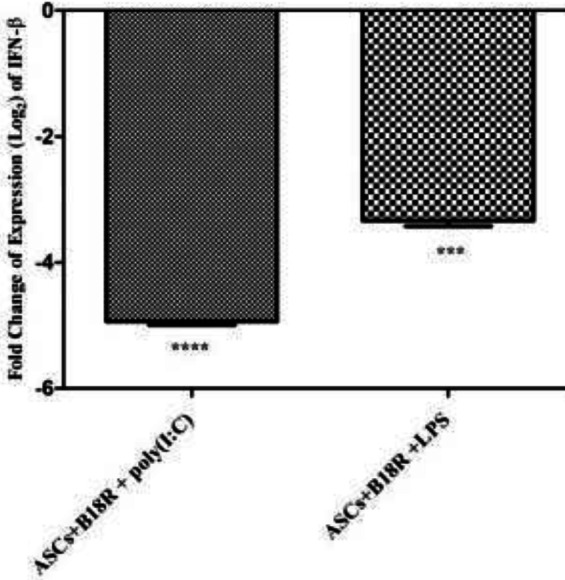
Gene expression analysis of the ASCs+B18R+poly(I:C) and LPS in comparison with these groups without b18R for *IFN-β*. The relative expression level of *IFN-β *has significantly decreased in former groups (*** *P*-value<0.001)

ASCs: Adipose-derived MSCs; LPS: lipopolysaccharide

Comparing the results, it was observed that during incubation with B18R, the expression of *IFN- β* was significantly decreased (Figure 7).

## Discussion

B18R is a highly specific cytokine receptor that binds to IFN-I and acts as an inhibitor. Besides the clinical application of this protein in an oncolytic virus structure, it is considered a great aid in technologies and research experiments. The oncolytic virus genome containing the B18R gene, with an antagonizing effect against IFN-I, can enhance the anti-tumor immunity of the harboring virus. Inhibition of immune response activation and degradation of exogenously delivered mRNA is provided by co-transfection of synthetic mRNA with B18R mRNA or incubation of targeted cells with recombinant B18R protein (1, 2, 5).

Thanks to the immunomodulatory and regenerative properties of MSCs, these cells and their products have many clinical applications. The determining criteria by the International Society for Cell and Gene Therapy (ISCT) for defining MSCs include the capacity to differentiate between a variety of cell types, plastic-adherent properties, and over-expression of some cell surface markers such as CD73, CD90, and CD105. To modify the MSCs toward improved phenotypes and therapeutic capacities, several optimized strategies have been widely used, most of which are based on preconditioning techniques (13). 

In stem cell-based therapy, many investigations have been undertaken on MSCs with a favorable immunosuppressing phenotype in immune-related inflammatory diseases. The most reliable strategy in cell-based therapy is autologous stem cells; however, some immune-related conditions are associated with impaired stem cells, such as deficient MSCs. Thus, considering the relatives of patients as donors is an alternative. On the other hand, in addition to different priming approaches to increase the proliferation and immunomodulatory properties in these cells, there are some recommended *in vivo* methods to improve the impaired properties and ameliorate the symptoms (14). MSCs are a group of cells in the bone marrow stroma and have a role in generating fibrosis in MPN patients suffering from over-expression of cytokines, including IL6 and MCP-1. The pretreatment of MSCs of these patients with Ruxo is associated with reducing these cytokines (10). SLE BMSCs have undergone senescence resulting from overexpression of IL6, IL8, MCP 2, and GM-CSF genes. Overproduction of IFN-β in MSCs of these patients and its target transcripts eventually causes inflammation, followed by cell senescence (7). This observation leads to the hypothesis that inhibition of this pathway may result in reduced inflammation.

A defective phenotype is observed in diabetic patients’ MSCs, resulting in complications in wound repair and reduced regenerative capacity. Shin and Peterson have shown that the expression of inflammatory factors, including IL6, TNF-a, and CXCL2, have increased in the diabetic wound environment. On the other hand, the impairment in Wnt3a expression in diabetic MSCs was associated with poor survival and low rate of proliferation. These deficiencies in the wound environment have resulted in delayed repair. A recommended advanced therapeutic strategy is the *in vivo* activation of MSCs toward increased proliferation (14).

Previous studies have suggested that activation of TLRs could affect the function of MSCs. Although contradictory results have been reported, several investigations have indicated that these receptors’ activation may increase the immune suppression capacity of MSCs. TLR3 and TLR4 activation results in overexpression of IDO in MSCs, which leads to inhibition of T cell proliferation (15). In an investigation into the role of poly(I:C) on MSCs function, it was demonstrated that poly(I:C)-treated MSCs were associated with enhanced production of soluble factors, including IL6, IL10, IL11, leukemia inhibitory factor (LIF), vascular endothelial growth factor (VEGF), and stromal cell-derived factor-1 (SDF-1). These changes finally led to the immunosuppressive properties of MSCs (16). A comprehensive study on gene expression changes in human MSCs primed with LPS demonstrated that TLR4-priming leads to increased expression of several genes associated with chemotaxis and inflammation, as well as significant increases in cytokines and chemokines. Transcriptome sequencing results showed that the expression level of several genes such as LIF, IL6, CCL2, CCL3, CCL5, CCL7, and IL1-α has increased after priming with LPS. Although the IFN signaling pathway was activated, the change in the expression of IFN-α /β genes did not appear in the RNA sequencing results (17). 

In the present study, we investigated the expression level of some immunity response-related genes in ASCs after priming with B18R. Firstly, following the addition of B18R to the culture media, TLR3 and TLR4-induced immunity activation was performed 24 hr later through the exposure of the cells to poly(I:C) and LPS, respectively. Then, the expression profile of several genes was provided via quantitative real-time PCR. The overexpression of TLR3 and TLR4 was observed in samples primed with their ligands compared with the cells without any treatment (as controls). Therefore, we concluded that TLR3 and TLR4 engagement would increase the expression of immunity response-related genes. Similar to Kim *et al*.’s study (1), the change in expression of IFN-β and IFN-γ was not detected via real-time PCR in these samples because the control samples showed no expression of these genes, and the treated groups were analyzed and compared with controls. Here, the expression of IFN-β and IFN-γ was induced in poly(I:C)- and LPS-treated ASCs after normalizing their expressions with RPLP0 as the interior control gene. As a result, *IDO1*, *TDO2*, *COX*2, *TGF-β1*, *HGF*, *IL1-β*, *IL6*, *MCP1* (*CCL2*), and *TNF-α* down-regulation was observed in B18R primed ASCs, which were functionally activated with poly(I:C) and LPS compared with the ASCs, which have been only treated with poly(I:C) and LPS. These genes were divided into two groups: immunomodulatory genes (*IDO1*, *TDO2*, *COX2*, *TGF-β1*, and *HGF*) and inflammatory cytokines genes (*IL1-β*, *IL6*, *MCP1*, and *TNF-α*). Although the expression of *IFN-β* and *IFN-γ* have increased after poly(I:C) and LPS treatment, they are under-expressed in the presence of B18R. 

It has been shown that cell injury caused upon treatment with LPS has been repaired after co-culture of the EpH4-Ev cells with MSCs overexpressing angiotensin-converting enzyme 2. The expression of inflammatory mediators including *TNF-α*, *IL-Iβ*, *IL-6,* and *iNOS* has down-regulated significantly following the interaction of modified MSCs with cells carrying LPS-induced inflammation (18). LPS-stimulated macrophages have been inhibited to produce pro-inflammatory cytokines, in presence of IL-4-treated MSCs. It is suggested that a therapeutic method can be the application of an anti-inflammatory agent to suppress the immune response of the immune cells which results from overproduction of pro-inflammatory cytokines (19). In the current study, B18R acts as an anti-inflammatory agent in suppressing the production of several pro-inflammatory cytokines.

Overall, for the first time, our findings reported an association between the effect of B18R in the environment of ASCs activated functionally with poly(I:C) and LPS and the expression level of some immunity response-related genes. We demonstrated that the genes, including *IDO1*, *TDO2*, *COX2*, *TGF-β1*, *HGF*, *IL1-β*, *IL6*, *MCP1*, and *TNF-α,* were significantly down-regulated in ASCs after preconditioning with B18R and stimulating with poly (I:C) and LPS. The clinical success of MSCs in regenerative and immunomodulatory treatments (20) has led to new approaches to isolation, expansion, and priming of these cells to improve treatment outcomes. Whether used *in vitro* or *in vivo*, B18R has been introduced as an altering agent that can affect the immunoregulatory properties of MSCs by suppressing IFNs. The functional activity, safety, and immunoregulatory capability of these primed cells remain to be explored with further studies.

**Table 1:** Primer sequences used for RT-qPCR

**Gene name** **(access number)**	**Primer sequence** **(5ʹ–3ʹ)**	**Product size** **(bp)**
*RPLP0* (NM_053275.4)	F: TGGTCATCCAGCAGGTGTTCGA R: ACAGACACTGGCAACATTGCGG	119
* IDO1*	F: TCATCTCACAGACCACAAGTCA	107
(NM_002164.6)	R: GCAAGACCTTACGGACATCTCC	
*TDO2* (NM_005651.4)	F: ACCTCCGTGCTTCTCAGACAG R: GACCTCCTTTGCTGGCTCTATTC	151
*COX-2* (NM_000963.3)	F:CCAGAGCAGGCAGATGAAATACC R: ACCAGAAGGGCAGGATACAGC	168
*TGF-β1* NM_000660.7	F: GTTCAAGCAGAGTACACACAGC R: GTATTTCTGGTACAGCTCCACG	154
*TNF-α* (NM_000594.4)	F: GCTGGTTATCTCTCAGCTCCA R: CTTCTCCTTCCTGATCGTGG	266
*IL-1β* (NM_000576.2)	F: CCTCTCTCACCTCTCCTACTCAC R: CTGCTACTTCTTGCCCCCTTTG	186
*IL-6* (NM_000600.4)	F: ACTCACCTCTTCAGAACGAATTG R: GCAAGTCTCCTCATTGAATCCAG	196
*TLR3* (NM_003265.3)	F: CAAACACAAGCATTCGGAATCTG R: AAGGAATCGTTACCAACCACATT	145
*TLR4* (NM_138554.5)	F: AGTTGATCTACCAAGCCTTGAGT R: GCTGGTTGTCCCAAAATCACTTT	94
*MCP-1* (NM_002982.4)	F: GCTCATAGCAGCCACCTTCATTC R: GGACACTTGCTGCTGGTGATTC	147
*HGF* (NM_001010931.3)	F: GCCATGAATTTGACCTCT R: GACATTTGATGCCACTCTTA	111

ASCs: Adipose-derived MSCs

## Conclusion

In this study, we investigated whether the expression of immunoregulatory genes is changed following the treatment of MSCs preconditioned B18R and then treated with poly(I:C) and LPS. We induced an inflammatory response in the AMSCs through preconditioning of these cells with the two mentioned stimuli and then checked the expression of some immune-related genes at the RNA level upon treatment of these cells with B18. Our results reveal that B18 leads to suppression of investigated immune-related genes which are overexpressed following immune response stimulation. These findings suggest that B18R can be considered a modulator of inflammatory response and further investigations need to be performed to check the changes at protein level of cytokines and other inflammatory mediators.

## Authors’ Contributions

HB, MF, and HR designed the experiments; HB, MF, MK, and HH performed the experiments and collected data; HH analyzed data; HB, MK, and HR prepared the draft of manuscript; HR supervised and directed the study. All the authors (HB, MF, MK, HH, and HR) approved the final version of the submitted manuscript. 

## Ethics Approval

Ethics committee approval of this study was obtained from ACECR-Khorasan Razavi Biomedical Research Ethics Committee (Code: IR.ACECR.JDM.REC.1398.007). 

## Conflicts of Interest

The authors declare that they have no competing interests.

## References

[1] Kim YG, Baltabekova AZ, Zhiyenbay EE, Aksambayeva AS, Shagyrova ZS, Khannanov R (2017). Recombinant Vaccinia virus-coded interferon inhibitor B18R: Expression, refolding and a use in a mammalian expression system with a RNA-vector. PLoS One.

[2] Michel T, Golombek S, Steinle H, Hann L, Velic A, Macek B (2019). Efficient reduction of synthetic mRNA induced immune activation by simultaneous delivery of B18R encoding mRNA. J Biol Eng.

[3] Colamonici OR, Domanski P, Sweitzer SM, Larner A, Buller RM (1995). Vaccinia virus B18R gene encodes a type I interferon-binding protein that blocks interferon alpha transmembrane signaling. J Biol Chem.

[4] Alcamí A SJ, Smith GL (2000). The vaccinia virus soluble alpha/beta interferon (IFN) receptor binds to the cell surface and protects cells from the antiviral effects of IFN. J Virol.

[5] Fu X, Rivera A, Tao L, Zhang X (2012). Incorporation of the B18R gene of vaccinia virus into an oncolytic herpes simplex virus improves antitumor activity. Mol Ther.

[6] Pittenger MF, Discher DE, Peault BM, Phinney DG, Hare JM, Caplan AI (2019). Mesenchymal stem cell perspective: Cell biology to clinical progress. NPJ Regen Med.

[7] Gomez-Salazar M, Gonzalez-Galofre ZN, Casamitjana J, Crisan M, James AW, Peault B (2020). Five Decades Later, Are Mesenchymal Stem Cells Still Relevant?. Front Bioeng Biotechnol.

[8] Noronha NC, Mizukami A, Caliari-Oliveira C, Cominal JG, Rocha JLM, Covas DT (2019). Priming approaches to improve the efficacy of mesenchymal stromal cell-based therapies. Stem Cell Res Ther.

[9] Gao L, Bird AK, Meednu N, Dauenhauer K, Liesveld J, Anolik J (2017). Bone marrow-derived mesenchymal stem cells from patients with systemic lupus erythematosus have a senescence-associated secretory phenotype mediated by a mitochondrial antiviral signaling protein-interferon-beta feedback loop. Arthritis Rheumatol.

[10] Zacharaki D, Ghazanfari R, Li H, Lim HC, Scheding S (2018). Effects of JAK1/2 inhibition on bone marrow stromal cells of myeloproliferative neoplasm (MPN) patients and healthy individuals. Eur J Haematol.

[11] Naderi-Meshkin H, Andreas K, Matin MM, Sittinger M, Bidkhori HR, Ahmadiankia N (2014). Chitosan-based injectable hydrogel as a promising in situ forming scaffold for cartilage tissue engineering. Cell Biol Int.

[12] Bidkhori HR, Ahmadiankia N, Matin MM, Heirani-Tabasi A, Farshchian M, Naderi-Meshkin H (2016). Chemically primed bone-marrow derived mesenchymal stem cells show enhanced expression of chemokine receptors contributed to their migration capability. Iran J Basic Med Sci.

[13] Yin JQ, Zhu J, Ankrum JA (2019). Manufacturing of primed mesenchymal stromal cells for therapy. Nat Biomed Eng.

[14] Peterson DA (2012). Impaired therapeutic capacity of autologous stem cells in a model of type 2 diabetes. Stem Cells Transl Med.

[15] Zhao X, Liu D, Gong W, Zhao G, Liu L, Yang L (2014). The toll-like receptor 3 ligand, poly(I:C), improves immunosuppressive function and therapeutic effect of mesenchymal stem cells on sepsis via inhibiting MiR-143. Stem Cells.

[16] Mastri M, Shah Z, McLaughlin T, Greene CJ, Baum L, Suzuki G (2012). Activation of toll-like receptor 3 amplifies mesenchymal stem cell trophic factors and enhances therapeutic potency. Am J Physiol Cell Physiol.

[17] Qiu Y, Guo J, Mao R, Chao K, Chen BL, He Y (2017). TLR3 preconditioning enhances the therapeutic efficacy of umbilical cord mesenchymal stem cells in TNBS-induced colitis via the TLR3-Jagged-1-Notch-1 pathway. Mucosal Immunol.

[18] Yan S, Ye P, Aleem MT, Chen X, Xie N, Zhang Y (2021). Mesenchymal stem cells overexpressing ace2 favorably ameliorate lps-induced inflammatory injury in mammary epithelial cells. Front Immunol.

[19] Jin QH, Kim HK, Na JY, Jin C, Seon JK (2022). Anti-inflammatory effects of mesenchymal stem cell-conditioned media inhibited macrophages activation in vitro. Sci Rep.

[20] Rodríguez-Fuentes DE, Fernández-Garza LE, Samia-Meza JA, Barrera-Barrera SA, Caplan AI, Barrera-Saldaña HA (2021). Mesenchymal stem cells current clinical applications: A systematic review. Arch Med Res.

